# Immunoprofiling of monocytes in STAT1 gain-of-function chronic mucocutaneous candidiasis

**DOI:** 10.3389/fimmu.2022.983977

**Published:** 2022-09-12

**Authors:** Marketa Bloomfield, Irena Zentsova, Tomas Milota, Anna Sediva, Zuzana Parackova

**Affiliations:** ^1^ Department of Immunology, 2^nd^Faculty of Medicine Charles University, University Hospital in Motol, Prague, Czechia; ^2^ Department of Paediatrics, Thomayer University Hospital, First Faculty of Medicine, Charles University, Prague, Czechia

**Keywords:** candidiasis, cmc, monocytes, dendritic cell, STAT1, ruxolitinib, immunodeficiencies

## Abstract

Patients with STAT1 gain-of-function (GOF) mutations suffer from an inborn error of immunity hallmarked by chronic mucocutaneous candidiasis (CMC). The pathogenesis behind this complex and heterogeneous disease is still incompletely understood. Beyond the well-recognized Th17 failure, linked to the STAT1/STAT3 dysbalance-driven abrogation of antifungal defense, only little is known about the consequences of augmented STAT1 signaling in other cells, including, interestingly, the innate immune cells. STAT1-mediated signaling was previously shown to be increased in STAT1 GOF CD14+ monocytes. Therefore, we hypothesized that monocytes might represent important co-orchestrators of antifungal defense failure, as well as various immunodysregulatory phenomena seen in patients with STAT1 GOF CMC, including autoimmunity. In this article, we demonstrate that human STAT1 GOF monocytes are characterized by proinflammatory phenotypes and a strong inflammatory skew of their secretory cytokine profile. Moreover, they exhibit diminished CD16 expression, and reduction of classical (CD14++C16-) and expansion of intermediate (CD14++16+) subpopulations. Amongst the functional aberrations, a selectively enhanced responsiveness to TLR7/8 stimulation, but not to other TLR ligands, was noted, which might represent a contributing mechanism in the pathogenesis of STAT1 GOF-associated autoimmunity. Importantly, some of these features extend to STAT1 GOF monocyte-derived dendritic cells and to STAT1 GOF peripheral myeloid dendritic cells, suggesting that the alterations observed in monocytes are, in fact, intrinsic due to STAT1 mutation, and not mere bystanders of chronic inflammatory environment. Lastly, we observe that the proinflammatory bias of STAT1 GOF monocytes may be ameliorated with JAK inhibition. Taken together, we show that monocytes likely play an active role in both the microbial susceptibility and autoimmunity in STAT1 GOF CMC.

## Introduction

Hypermorphic mutations in signal transducer and activator of transcription 1 gene (STAT1 gain-of-function, STAT1 GOF) in humans cause an inborn error of immunity which is hallmarked by chronic mucocutaneous candidiasis (CMC). This complex syndrome of immune dysregulation displays a broad phenotype beyond the microbial susceptibility, including autoimmunity (often severe and life threatening), malignancy and vascular abnormalities (1–4). While the increased fungal susceptibility is now well linked to the failure of Th17 immunity (5), the non-infectious features of STAT1 GOF CMC are much less understood. JAK inhibition (with compounds ruxolitinib or baricitinib) have recently proved effective in ameliorating both the candida susceptibility and the autoimmunity in some patients with STAT1 GOF mutations (6, 7).

The Janus Activating Kinase (JAK)/STAT pathway is utilized by both adaptive and innate immune cells in response to signals from many various cytokines and hormones. It encompasses seven structurally homologous STAT proteins which are involved in a range of essential cellular processes, such as growth, differentiation, metabolism and immune functions (8–10). STAT1 transduces signals from interferons and IL-27, promoting the expression of interferon-stimulated genes (11). In the cells of STAT1 GOF patients (as well as in STAT1 GOF cell models), increased STAT1 activity is proposed to be caused by one, or a combination of the following: augmented STAT1 phosphorylation, delayed STAT1 dephosphorylation, increased total STAT1 protein levels or increased stability of the STAT1 dimer (12, 13). These events likely result in altered histone acetylation upon STAT1-DNA interaction. Consequently, the transcription of STAT3-inducible genes is reduced, as STAT1 and STAT3 compete for the DNA-binding sites. Thus, the STAT3-mediated Th17 differentiation is hampered, and the Th17-dependent defense against *Candida* is impaired (5, 14, 15).

Although more than 100 STAT1 GOF mutations in over 400 patients have been identified worldwide to date (16), only two studies (suggesting the functional alteration of natural killer cells) have addressed the possible involvement of innate immune cells in the disease, beyond the description of increased pSTAT1 (17, 18). Monocytes are innate immune cells involved not only in the first-line antimicrobial host defense, but also in the initiation, mediation, and regulation of inflammatory processes, as well as in the tissue repair and homeostatic processes (19). This cell lineage is derived from bone marrow from a common precursor shared with macrophages and dendritic cells (DCs), into which monocytes may also differentiate directly. Three distinct subsets of monocytes are recognized: the classical (CD14++ CD16-), intermediate (CD14++ CD16+) and nonclassical (CD14+CD16++). These subsets differ in numbers, blood/tissue distribution, and functional properties (20). Interestingly, monocytes have been associated with various autoimmune diseases or immunodysregulatory phenomena (21) and previous research showed that STAT1 GOF mutations affect the JAK/STAT1-mediated signaling in CD14+ monocytes (1, 3, 13, 22). Therefore, we hypothesized that monocytes might play an active role in the pathogenesis of immune dysregulation in STAT1 GOF CMC, perhaps even beyond the fungal susceptibility. In this report, we set out to explore the phenotype and functions of human *ex-vivo* STAT1 GOF monocytes.

## Methods

### Patients

**Figure 1:**
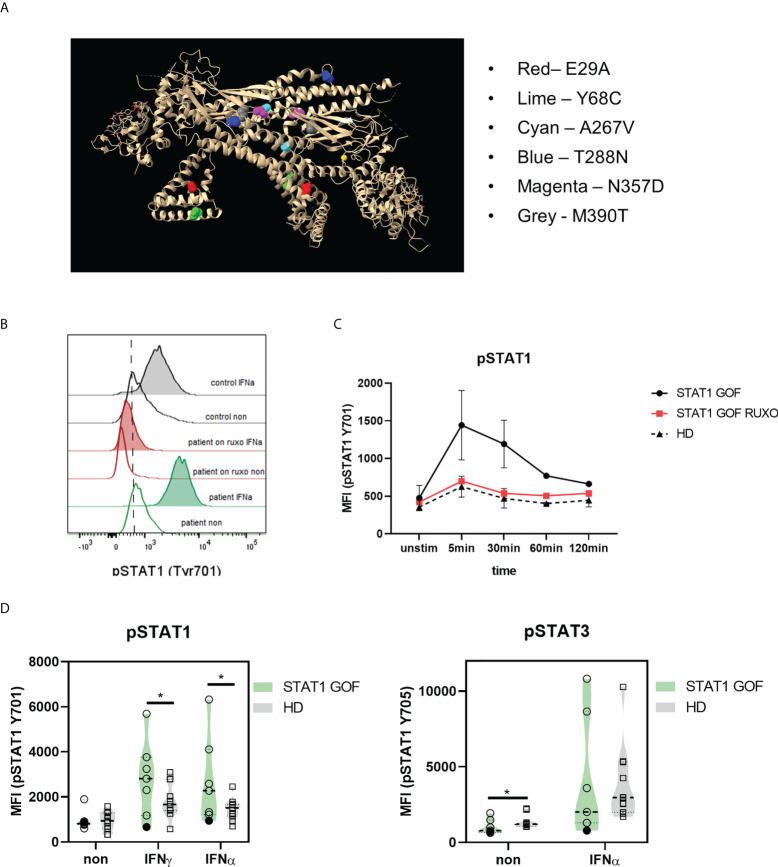
STAT1 mutations in the patient cohort. **(A)** STAT1 mutations in the 3D dimeric protein structure; the position of each mutation is highlighted **(B)** Representative histogram of STAT1 phosphorylation (Tyr701) upon IFNα stimulation in STAT1 GOF and HDs’ monocytesdetected by flow cytometry **(C)** Kinetics of STAT1 and STAT3 phosphorylation (Tyr701) upon IFNα stimulation in STAT1 GOF (n = 3) and HDs’ monocytes (n = 4) detected by flow cytometry **(D)** Phosphorylation of STAT1 (pSTAT1; Tyr701) and STAT3 (pSTAT3; Tyr705) upon IFNγ and IFNα stimulation in STAT1 GOF (n = 7) and HDs’ monocytes (n = 11) detected by flow cytometry HD - healthy donors; Tyr - tyrosine. Values are standardized and expressed as median values. Statistical analyses were performed using paired t-tests. Values of p<0.05 (*), p<0.01 were considered statistically significant.

The biologic material was obtained from 8 Czech patients followed at the Department of Immunology, 2nd Faculty of Medicine, Charles University and University Hospital in Motol, who were diagnosed with CMC due to *STAT1* mutation. All patients were non-consanguineous, of Caucasian ethnicity, 3 males and 5 females, the median age was 45 years (range 8–52 years). All patients suffered from CMC of various severity, three had clinically manifest autoimmunity and six had autoantibodies against organ-nonspecific antigens. All patients received antifungal prophylaxis. Three patients received selective Janus kinases (JAK) 1/2 inhibitor ruxolitinib (one each for CMC and severe autoimmune anemia, refractory CMC and severe lung disease, and CMC and severe keratitis, respectively, all of which improved on the treatment), however majority of samples for this study were obtained prior to ruxolitinib initiation, unless indicated otherwise. No patients received any other immunomodulatory compounds at the time of sampling or during a relevant period prior to sampling. The mutations within the cohort include previously described heterozygous mutations causing amino acid change in p.E29A, p.Y68C, p.A267V, p.T288N, p.N357D and p.M390T (**Figure 1A**). Consistently with STAT1 GOF immunophenotype, all patients had increased IFNα- and/or IFNγ-induced STAT1 signaling in CD3+ T cells and low numbers of peripheral CD4+ Th17 cells. Monocyte counts were within age-matched reference ranges in all patients (**Supplementary Table**). Corresponding age and sex-matched healthy donors (HD) were enrolled into the study. The study was carried out in accordance with the recommendations of the Ethical Committee of the second Faculty of Medicine, Charles University in Prague, and University Hospital in Motol, Czech Republic. The study protocol was approved by the institutional Ethical Committee. All subjects or their legal guardians gave written informed consents with the research and publication in accordance with the Declaration of Helsinki.

### Phosphoflow

Whole blood was stimulated with 1µg/ml IFNγ or IFNα (Abcam, Cambridge, UK) for 5, 15, 30, 60 and 120 minutes or left untreated at 37°C. Intracellular signaling was prevented by using 4% paraformaldehyde without methanol for 10 minutes at room temperature. Erythrocytes were lysed using 0,1% Triton-X for 20 minutes (Sigma Aldrich, St. Luis, USA) at 37°C, leukocytes were permeabilized with ice-cold 80% methanol for 30 minutes and stained with anti-phosphoSTAT1-BV421 (Tyr701) (clone 4a) and anti-phosphoSTAT3-PE (Tyr705) (clone 4/5-STAT3) (both from BD Bioscience, San Jose, USA), anti CD14-APC (63D3), CD66b-PC7 (clone G10F5) (BioLegend, San Diego, USA) in case of whole blood. The samples were acquired on BD Fortessa (BD Biosciences), and data analysis was performed using FlowJo (TreeStar).

### Monocyte and DC subsets

Monocytes and dendritic cells from peripheral blood were stained with the following antibodies: Lin-FITC (CD3, CD19, CD20 and CD56), CD16-A700 (clone 3G8), CD11c-APC (clone BU15), CD14-PE-DyLight594 (clone MEM-15) CD5-APC (L17F12) (Exbio), HLA-DR-PerCP (clone L243) (BD Biosciences), CD123-PE-Cy7 (clone 6H6), CCR2-BV421 (clone K036C2), CD1c-BV510 (clone L161), CD141-BV421 (clone M80) CD163-BV510 (GHI/61) (Biolegend), for 30 minutes in the dark. The cells were then lysed, centrifuged, resuspended and data were acquired on BD Fortessa (BD Biosciences), and data analysis was performed using FlowJo (TreeStar).

### Monocyte and DC phenotype

Monocytes and dendritic cells from peripheral blood were stained with the following antibodies: Lin-FITC (CD3, CD19, CD20 and CD56), CD16-A700 (clone 3G8), CD11c-APC (clone BU15), CD14-PE-DyLight594 (clone MEM-15), CD86-PE (clone BU63) (Exbio), HLA-DR-PerCP (clone L243) (BD Biosciences), PD-L1-BV510 (clone 29E.2A3), PD-1-BV421 (clone EH12.2H7), CD123-PE-Cy7 (clone 6H6), CD40-BV650 (clone 5C3) (Biolegend) for 30 minutes in the dark. The cells were then lysed, centrifuged, resuspended and data were acquired on BD Fortessa (BD Biosciences), and data analysis was performed using FlowJo (TreeStar).

### Monocyte and DC maturation

Peripheral blood was stimulated with LPS (1ug/ml), zymosan (100ng/ml), R848 (1ug/ml), polyI:C (50ug/ml), IFNγ (1ug/ml), heat-killed 1x10^6^ *C. albicans *or left untreated for 24 hours. Then CD86 expression was detected by staining the peripheral blood with Lin-FITC (CD3, CD19, CD20 and CD56), CD16-A700 (clone 3G8), CD11c-APC (clone BU15), CD14-PE-DyLight594 (clone MEM-15), CD86-PE (clone BU63), HLA-DR-PerCP (clone L243) (BD Biosciences), CD123-PE-Cy7 (clone 6H6), CD1c-BV510 (clone L161) and CD141-BV421 (clone M80) (Biolegend) for 30 minutes in the dark. The cells were then lysed, centrifuged, resuspended and data were acquired on BD Fortessa (BD Biosciences), and data analysis was performed using FlowJo (TreeStar).

### Cytokine production

For intracellular cytokine detection, 100ul of peripheral blood from heparin coated tubes was stained against lineage specific markers (CD3, CD19, CD20, CD56) conjugated with FITC, CD16-Alexa Fluor 700, CD11c-APC, CD14-PE-DyLight594 (Exbio), HLA-DR-PerCP (BD Biosciences), CD1c-BV510, CD141-BV421 (BioLegend), and CD123-PE-Cy7 (ThermoFisher Scientific). After RBC were lysed with BD Lysing solution (BD Biosciences), cells were fixed and permeabilized using FixPerm kit (ThermoFisher Scientific). Cytokines were stained using anti IL-6-PE (clone MQ2-13A5) (Biolegend), IL-1β-PE (clone CRM56) – (ThermoFisher Scientific) TNFα-PE (clone MAb11) (Exbio) or CXCL10-PE (clone J034D6), respectively. When indicated, peripheral blood was stimulated with R848 (1ug/ml) for 4 hours and 1 μl/ml Brefeldin A (Biolegend) was added for the last 3 h of the incubation (BD Biosciences). CXCL10 production was detected as described above.

### CD16 expression

For CD16 extracellular detection, whole blood was stained with the following antibodies: Lin-FITC (CD3, CD19, CD20 and CD56), CD16-A700 (clone 3G8), CD11c-APC (clone BU15), CD14-PE-DyLight594 (clone MEM-15) (Exbio), CCR2-BV421 (clone K036C2) (Biolegend), HLA-DR-PerCP (clone L243) (BD Biosciences). For intracellular CD16 detection the whole blood was lysed, fixed and permeabilized before CD16-A700 staining. Data was acquired on BD Fortessa (BD Biosciences), and data analysis was performed using FlowJo (TreeStar).

### Generation of monocyte-derived dendritic cells

Monocyte-derived DCs (cDCs) were generated from adherent monocytes cultured in IL-4 (20ng/mL) and GM-CSF (500IU/mL) (CellGenix, Freiburg, Germany) presence for 6 days. The cytokines were replenished on day 3. On day 6, the cells were harvested, seeded in 96-well plates at 1x10^6^/mL concentration and the next day the phenotype and cell-free supernatant was collected for cytokine production assessment. Cells were stained with anti, CD14-PEDy590 (clone MEM15), CD86-PerCP (clone BU63), CD16-A700 (clone 3G8) (Exbio), HLA-DR-A700 (clone L243), CD40-BV650 (clone 5C3), PD-L1-BV510 (clone 29E.2A3) (BioLegend). The samples were collected using BD Fortessa (BD Biosciences) and BD FACSDiva software (BD Biosciences) was used for signal acquisition. Cytokine production was determined byLuminex xMAP technology.

### Serum cytokine/chemokine/growth factors levels

Peripheral blood was collected into blood tubes without anticoagulant agent and centrifuged at 3000rpm, 5 minutes. The serum was collected, aliquoted and stored at 80°C. The serum cytokine, chemokine and growth factor quantification were performed by LUMINEX xMAP technology.

### Statistical analysis

The results obtained from at least three independent experiments are given as the medians ± SDs. Not all patients were involved in all experiments due to the limited amount of blood available per sample. Statistical analysis was performed using non-parametric one-way analysis of variance (ANOVA) with multiple comparisons Dunn’s post-test where applicable. A two-tailed paired Wilcoxon or unpaired Mann-Whitney *t*-test was also applied for data analysis using GraphPad Prism 8. Values of p<0.05 (*), p<0.01 (**) p<0.001 (***) and p<0.0001 (****) were considered statistically significant.

## Results

### STAT1 phosphorylation is increased in STAT1 GOF monocytes and reversible with ruxolitinib

Firstly, STAT1 phosphorylation assays in monocytes after IFNα and IFNγ stimulation were performed. As expected, most of the patients displayed increased levels of phosphorylated STAT1 (pSTAT1), except for patients carrying p.Y68C mutation (**Figures 1B, D**). In STAT1 GOF patients treated with ruxolitinib, the pSTAT1 was reduced to HDs’ levels. Regarding pSTAT1 kinetics, STAT1 GOF patients’ monocytes initiated the pSTAT1 dephosphorylation after similar time as HDs (5-30 minutes post stimulation) but took significantly longer than HDs to revert to basal unstimulated levels of pSTAT1 (**Figure 1C**), implying a delayed deactivation of the overt pSTAT1 activity. Patients treated with ruxolitinib exhibited similar kinetics as HDs (**Figure 1C**).

### Peripheral STAT1 GOF monocytes exhibit pro-inflammatory phenotype

**Figure 2:**
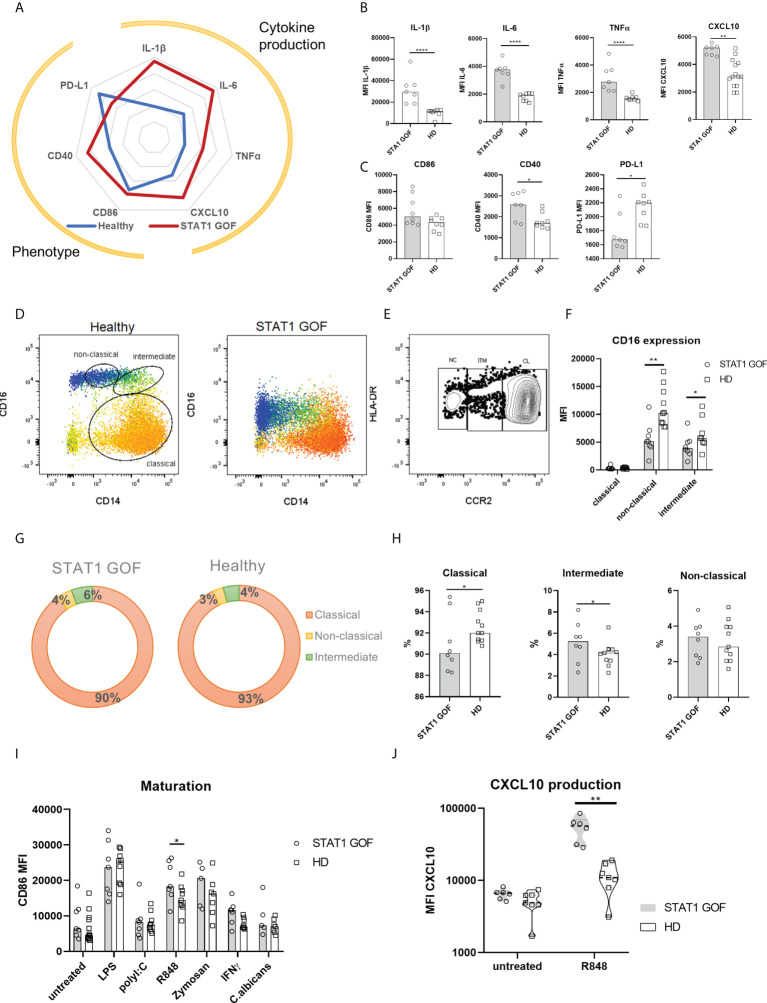
Monocyte characteristics. **(A)** Radar graph of STAT1 GOF (n = 7) and HDs’ (n = 8) monocyte phenotype and basal cytokine production **(B)** Cytokine production of STAT1 GOF (n = 7) and HDs’ (n = 8) monocytes detected by flow cytometry **(C)** Phenotype of STAT1 GOF (n = 7) and HDs’ (n = 8) monocytes detected by flow cytometry **(D)** Representative dot plot of monocyte subset determination **(E)** Representative dot plot of monocyte subset determination utilizing CCR2 expression **(F)** CD16 expression on monocyte subsets surface of STAT1 GOF (n = 7) and HDs (n=8) detected by flow cytometry **(G)** Distribution of monocyte subsets in STAT1 GOF (n = 7) and HDs (n = 8) **(H)** Quantification of monocyte subsets in STAT1 GOF (n = 7) and HDs (n = 8) **(I)** CD86 expression on monocyte surface upon stimulation of various TLR ligands for 24hours in STAT1 GOF (n=7) and HDs (n=9) detected by flow cytometry **(J)** CXCL10 production by monocytes upon R848 (1ug/ml) stimulation in STAT1 GOF (n = 7) and HDs (n=8) detected by flow cytometry HD - healthy donors. Values are standardized and expressed as median values. Statistical analyses were performed using paired t-tests. Values of p<0.05 (*), p<0.01 (**), and p<0.0001 (****) were considered statistically significant.

When STAT1 GOF CD14+ monocytes’ basal cytokine production and cellular phenotypes were assessed, a marked pro-inflammatory skew was noted (**Figures 2A–C**). Significantly higher production of IL-1β, IL-6, TNFα and CXCL10 by unstimulated STAT1 GOF monocytes was observed compared to HDs (**Figures 2A, B**). In parallel, enhanced expression of CD40 maturation marker and decreased expression of inhibitory PD-L1 surface molecules were detected in STAT1 GOF monocytes (**Figures 2A, C**). On the other hand, no difference in the expression of CD86, a classic monocyte inflammation marker, was observed.

### CD16 expression is critically diminished on STAT1 GOF monocytes

When examining the STAT1 GOF monocyte subsets distribution, the commonly used differentiators of the classical (CD14++CD16-), intermediate (CD14++CD16+) and non-classical (CD14+CD16++) monocytes (20) were indiscriminate due to very low CD16 expression (**Figure 2D**). Therefore, CCR2 expression was used to distinguish between the monocytes’ subsets (23) (**Figure 2E**). We observed diminished CD16 expression on both intermediate and non-classical monocytes (**Figure 2F**). Even though the data points distribution in our cohort was wide-ranging, a mild skew in STAT1 GOF monocytes’ subsets could be discerned (**Figures 2G, H**), characterized by a decrease of classical and increase of intermediate monocytes. Moreover, when CD16 was stained intracellularly, only a minimal difference was observed between STAT1 GOF and HDs’ monocytes, suggesting that CD16 internalization is not the cause of diminished CD16 surface expression in patients’ monocytes (**Supplementary Figure 1**).

### The responsiveness of STAT1 GOF monocytes to TLR7/8 stimulation is enhanced

Upon stimulation of STAT1 GOF monocytes with various Toll-like receptors (TLR) ligands, increased response was observed to R848 (imidazoquinoline), a dual TLR8 and TLR7 synthetic agonist, but not to lipopolysaccharide (LPS), polyI:C, fungal zymosan or *Candida* antigens. TLR7/8-stimulated STAT1 GOF monocytes expressed increased surface levels of CD86 and produced higher amounts of CXCL10 (**Figures 2I, J**), implying the involvement of STAT1 in TLR7/8-mediated inflammatory response.

### Myeloid DCs and their DC4 subsets are increased and plasmacytoid DCs are decreased in STAT1 GOF patients

**Figure 3:**
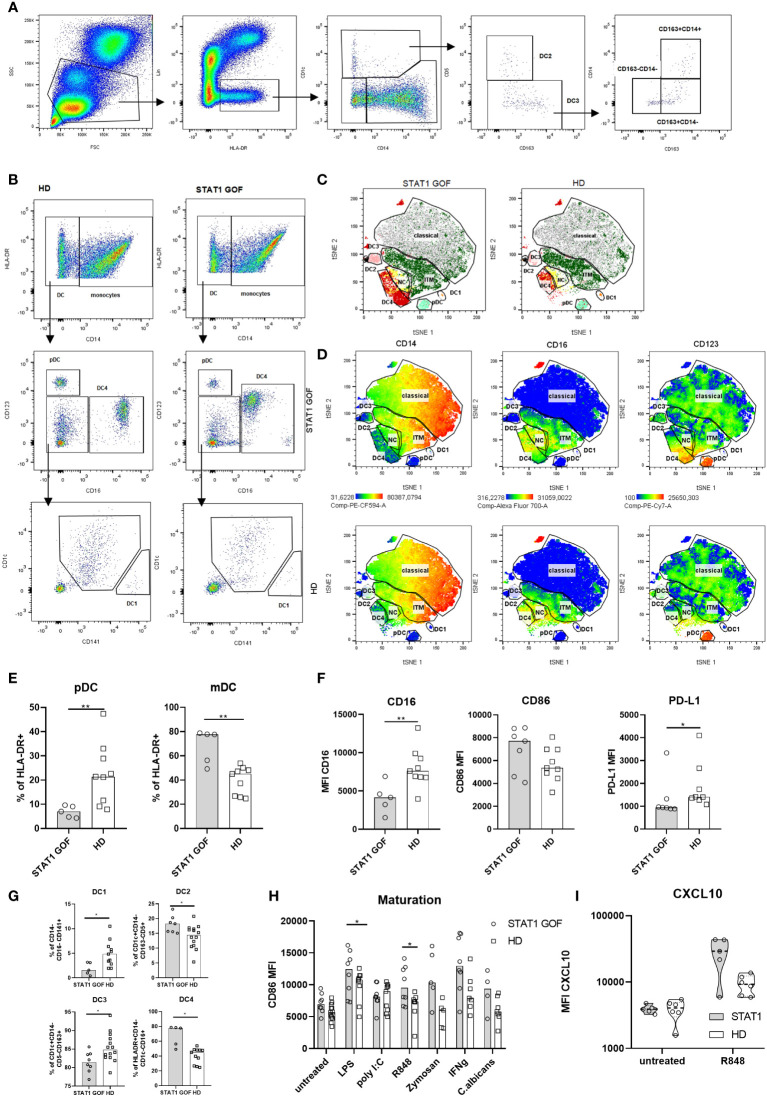
Dendritic cells characteristics **(A)** Gating strategy for DC2 and DC3 subsets analysis **(B)** Gating strategy forDC1 and DC4 determination **(C)** Color-coded gating strategy identifying DC subsets in STAT1 GOF patients (n = 5) and HDs (n = 5). The individual subsets are depicted as manually gated populations overlaid onto the tSNE plots **(D)** Representative tSNE plots of 5 STAT1 GOF and 5 HDs’ DC showing expression of CD14, D16 and CD123. The DC’s subsets are depicted as manually gated populations overlaid onto the tSNE plots **(E)** pDC and mDC quantification in STAT1 GOF (n = 7) and HDs (n = 9) detected by flow cytometry **(F)** mDC phenotype of STAT1 GOF (n = 7) and HDs’ DC (n = 9) detected by flow cytometry**(G)** Quantification of DCs’ subsets in STAT1 GOF and HDs detected by flow cytometry **(H)** CD86 expression on mDCs’ surface upon stimulation with various TLR ligands for 24hours in STAT1 GOF (n = 7) and HDs (n = 9) detected by flow cytometry **(I)** CXCL10 production by mDCs upon R848 (1ug/mL) stimulation in STAT1 GOF (n=7) and HD (n=8) detected by flow cytometry DC, dendritic cell; HD, healthy donors; tSNE, t-distributed stochastic neighbor embedding. Values are standardized and expressed as median values. Statistical analyses were performed using paired t-tests. Values of p<0.05 (*), and p<0.0001 (****) were considered statistically significant.

Bone marrow-derived monocytes are precursors of myeloid DCs, which constitute the majority of blood DCs in the healthy. As such, circulating DCs should also exhibit signs of dysregulation inflicted by STAT1 GOF mutation. Multiple subsets of DCs are recognized, each defined by unique combination of their surface markers. These include DC1 (CD14-CD1c-CD141+CD16-), DC2 (CD14-CD1c+CD5+CD163-), DC3 (CD14-CD1c+CD5-CD163+) and DC4 (CD14-CD1c-CD141-CD16+) as subtypes of mDCs (CD11c+HLA-DR+CD14-), and plasmacytoid DCs (pDCs; CD123+HLA-DR+CD14-) (24, 25). The gating strategies are illustrated in **Figures 3A, B**. Increased mDCs and decreased pDCs were found in the STAT1 GOF samples (**Figures 3C–E**). Also, several differences were revealed in the distribution of mDCs subpopulations, specifically increased DC2 and DC4 subsets and decreased DC1 and DC3 subsets (**Figures 3C, G**). Interestingly, STAT1 GOF DC4 also expressed increased levels of CD123 (**Figure 3D**). Similarly to monocytes, STAT1 GOF mDCs expressed lower levels of CD16 and PD-L1. We also observed higher levels of CD86, however, the result was not statistically significant (**Figure 3F**).

### The responsiveness of STAT1 GOF mDCs to TLR7/8 stimulation is enhanced

In a similar experimental setup as above, we examined mDCs’ responsiveness to various TLR ligands (**Figure 3H**). A higher reactivity was observed to LPS and R848. Upon stimulation, STAT1 GOF mDCs expressed increased surface levels of CD86, indirectly indicating a role of STAT1 in TLR4 and TLR7/8-induced inflammatory processes. Even though the CXCL10 production after R848 stimulation seemed to be increased in STAT1 GOF patients, it was statistically not significant (**Figure 3I**).

### Monocyte-derived DC model corroborates inflammatory profile of STAT1 GOF monocytes and DCs

**Figure 4:**
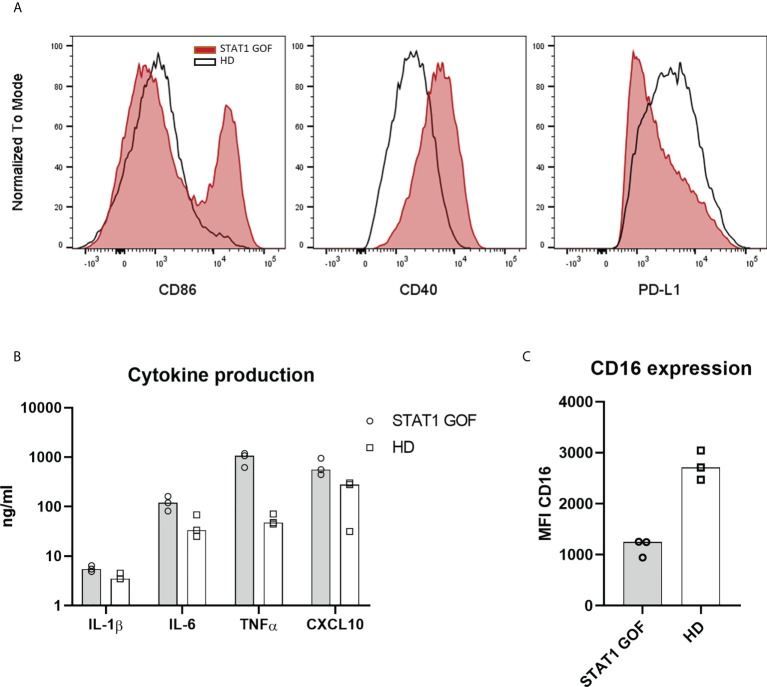
Monocyte-derived derived dendritic cells model **(A)** Expression of CD86, CD40 and PD-L1 on moDCs’ surface of STAT1 GOF (n = 3) and HDs (n = 3) detected by flow cytometry **(B)** Cytokine production of STAT1 GOF (n = 3) and HDs’ (n = 3) moDCs detected by LUMINEX **(C)** CD16 expression on moDCs’ surface of STAT1 GOF (n = 3) and HDs (n = 3) detected by flow cytometry.

To ascertain that the changes in monocytes and DCs are genotype-driven and not secondary to chronic infections, inflammation or treatment, an *in-vitro* monocyte-derived DC model (moDCs) was employed. After six days of cultivation from adherent monocytes, the generated STAT1 GOF moDCs expressed higher levels of CD86, CD40 and reduced amounts of PD-L1 **Figure 4A**, similarly to primary monocytes and DCs. STAT1 GOF moDCs produced increased levels of pro-inflammatory cytokines, such as IL-1β, IL-6, TNFα and CXCL10 (**Figure 4B**). Moreover, decreased expression of CD16 on the STAT1 GOF moDCs’ surface was observed, corresponding to the STAT1 GOF intermediate and non-classical monocytes (**Figure 4C**).

### Serum cytokines, chemokines and growth factors profiles are altered in STAT1 GOF patients

**Figure 5:**
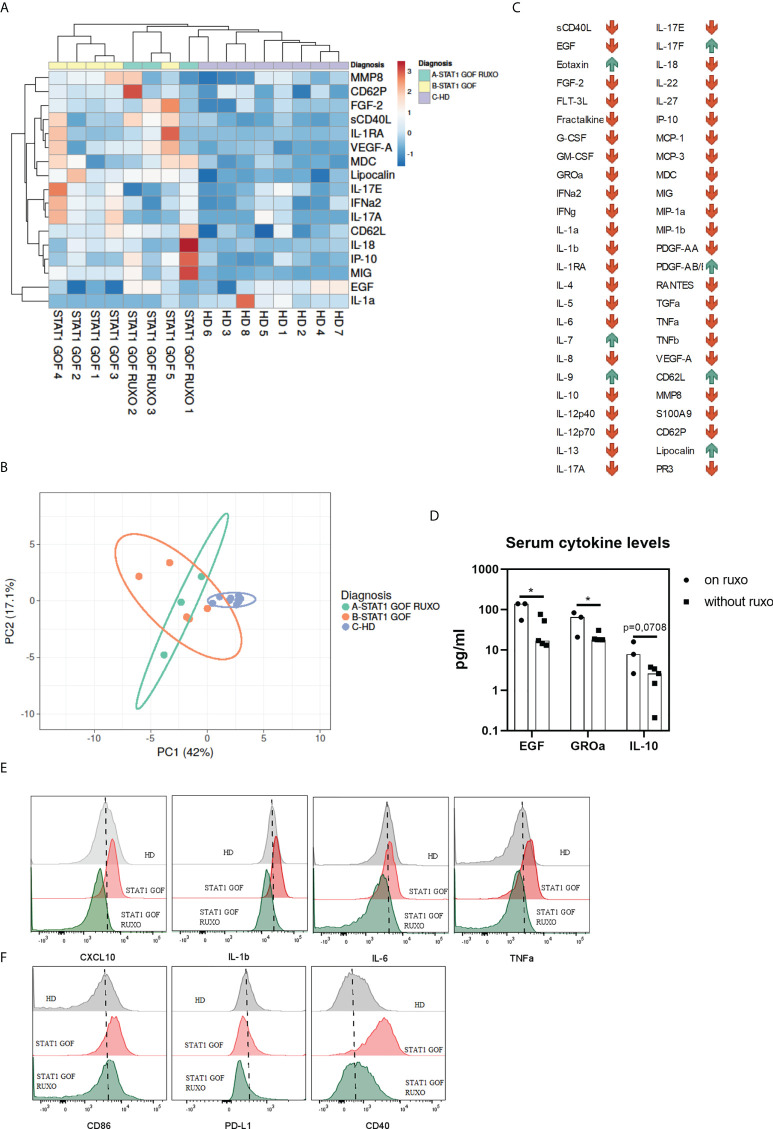
Serum cytokines/chemokines/growth factors **(A)** Heat map showing differences in serum levels of various cytokines, chemokines and GFs between STAT1 GOF patients (n = 5), ruxolitinib-treated STAT1 GOF patients (STAT1 GOF RUXO) (n = 3) and HDs (n = 7) detected by LUMINEX **(B)** Principal component analysis showing differences in serum levels of various cytokines, chemokines and GFs between STAT1 GOF patients (n=5), ruxolitinib-treated STAT1 GOF patients (STAT1 GOF RUXO) (n = 3) and HDs (n = 7) **(C)** Dynamics of analytes in a patient before and on ruxolitinib treatment **(D)** Differences in serum cytokine levels between ruxolitinib treated STAT1 GOF patients (n = 3) and untreated patients(n = 5) **(E)** Comparison of basal cytokine production by monocytes in HD, STAT1 GOF patient and the ruxolitinib-treated patient **(F)** Monocyte’s phenotype in HD, STAT1 GOF patient and the ruxolitinib-treated patient detected by flow cytometry HD - healthy donors; GF - growth factor. Values are standardized and expressed as median values. Statistical analyses were performed using paired t-tests. Values of p<0.05 (*), were considered statistically significant.

Monocytes are major producers of various stimulatory, pro-migratory cytokines and growth factors. Having previously established changes in STAT1 GOF mutant monocytes’ phenotype and secretory activities, we set out to explore the patients’ serum cytokine profiles. Out of 57 cytokines, chemokines and growth factors (GF) six were excluded: four (IL-2, IL-3, M-CSF and IFNβ) were undetectable and two (lactoferrin and CXCL4) exceeded the detection maximum. Out of 51 analytes, 17 cytokines/chemokines/GFs were differentially produced in the STAT1 GOF group compared to HDs (**Figures 5A, B**). These included cytokines involved in monocyte/macrophage biology, such as IL-1α, IL-1RA, CXCL10, IL-18, MDC, as well as in neutrophil functions, such as MMP8 and lipocalin. Interestingly, higher levels of IL-17A and IL-17E were detected in the STAT1 GOF patients’ sera.

When sera of STAT1 GOF patients treated with ruxolitinib were examined, 11 cytokines were produced differentially (lipocalin, sCD40L, VEGF-A, FLT-3L, MIG, IL-10, CD62L, MDC, IL-1RA, IL-10 and IL-9) compared to HDs (**Figure 5A**). Principal component analysis revealed that HDs separated distinctly from both patients’ groups (**Figure 5B**), suggesting that ruxolitinib treatment had only a minor effect on the serum cytokine/chemokine/GF profile in the patients. Indeed, two analytes only (EGF and GROα) differed statistically significantly between treated and untreated patient groups (**Figure 5D**).

However, when the serum cytokine profiles were sequentially analyzed in a patient before and on ruxolitinib (STAT1 GOF5 and STAT1 GOF RUXO1 in **Figure 1A**), the majority of analytes decreased on treatment, while few, including IL-17F, eotaxin, IL-7, IL-9, PDGF-AB/AA and lipocalin increased (**Figure 5C**).

### Ruxolitinib treatment partially ameliorated monocyte inflammatory bias

To assess whether the JAK/STAT signaling blockade would ameliorate the STAT1 GOF-driven inflammatory bias in monocytes, the phenotype and ligand-independent cytokine production of monocytes from a patient treated with ruxolitinib for over one year were analyzed. Compared to untreated patient’s monocytes, the ruxolitinib treated patient’s cells displayed reduced production of proinflammatory cytokines (**Figure 5E**) and reduced expression of CD86, CD40 and PD-L1 (**Figure 5F**).

## Discussion

In this article, we demonstrate that human peripheral monocytes harboring heterozygous hypermorphic mutations in *STAT1* gene are intrinsically altered in their phenotypes and functions. Characterized by diminished CD16 expression, reduction of classical (CD14++C16-) and expansion of intermediate (CD14++16+) subpopulations, monocytes adopt proinflammatory phenotypes and exhibit a strong inflammatory skew within their secretory cytokine profile. We also describe a selective enhancement of TLR7/8 responsiveness. Importantly, these features extended to STAT1 GOF monocyte-derived dendritic cells, and some to blood myeloid dendritic cells, which differentiate from monocytes. Finally, in one patient, from whom sequential samples were available, upstream JAK inhibition ameliorated the proinflammatory bias of STAT1 GOF monocytes.

STAT1 is an essential signaling component in monocytes’ biologic processes (26, 27). The consequences of its disbalanced actions in this cell line are not recognized in detail, however, they are likely multifaceted, context-dependent and heavily contraregulated. Monocytes of STAT1 GOF patients were originally shown to have increased levels of stimulation-induced tyrosine phosphorylated STAT1 protein (pSTAT1) (1, 3, 22). More recent studies declared that the reason for increased pSTAT1 levels may, in fact, be the increased total STAT1 protein (13, 28). In this study, increased levels of pSTAT1 and upregulation of STAT1-targeted genes, such as *CXCL10*, as well as CXCL10 serum increase were detected in STAT1 GOF patients’ monocytes and DCs, in concordance with others (29), the total STAT1 was not analyzed.

Monocytes represent a frontline innate cellular barrier against fungi. They have been associated with systemic candidiasis, specifically *via* their surface C-type lectin-like receptors Dectin 1 and Dectin 2 (30). It was also shown that both the classical and CD16+ monocytes can internalize and eradicate *Candida*, but only the classical CD16- monocytes promote the induction of Th17 and Th17-mediated responses to *Candida albicans* (31) (30). In our cohort, the diminished proportion of classical monocytes, favoring the intermediate subsets, was detected, which may, therefore, represent a contributing factor in the fungal defense failure.

The hereby observed loss of surface CD16 from monocytes, mDCs and moDCs is unclear as to its origin and significance. CD16 is a transmembrane Fc gamma type III low-affinity receptor for IgG expressed predominantly on natural killer cells, neutrophils, monocytes, macrophages and a subset of T cells. It is associated with phagocytosis, secretion of inflammatory cytokines and clearance of immune complexes (32). A well-controlled expression of CD16 is likely an important regulatory mechanism of cellular executive functions. Several possible mechanisms may be involved in its surface loss - increased internalization, degradation, excessive shedding or downregulation of CD16 expression. We observed only a small decrease in intracellular levels of CD16, suggesting that neither internalization, nor increased degradation are the likely culprits. CD16 shedding was shown to be a regulatory mechanism in natural killer cells, resulting in decreased immune cell contacts *via* immune synapses (33). Moreover, stimulation of monocytes with TLR agonists resulted in metalloproteinase ADAM17-mediated shedding of CD16+ from monocytes (34). Downregulation of CD16 has been previously described in natural killer cells after exposure to influenza vaccine, and, interestingly, the recovery of CD16 expression was surprisingly slow; in fact, CD16 only partially recovered by day 18 from the downregulation induction (35). Previous authors also revealed that CD16 regulates the TRIF-dependent TLR4 pathway in monocytes by activating STAT1 (and other proteins), which resulted in enhanced expression of CXCL10 (36). It is therefore conceivable that a negative feedback loop might exist, in which a constitutively overactivated STAT1 signaling would cause downregulation of CD16.

Monocytes have been associated with autoimmunity (37) and autoimmune phenomena represent frequent complications in patients with STAT1 GOF mutations. Although several hypotheses have been suggested, such as aberrant functions of regulatory T cells, B cells, and the upregulated type I IFN signaling (2, 38–41), the molecular mechanism behind autoimmunity in STAT1 GOF patients remains unexplained. It is most likely orchestrated by multiple elements. In this study, we challenged STAT1 GOF monocytes with various TLR agonists and noted augmented responsiveness to TLR7/8 ligand only. This is interesting as the receptor recognizes not only pathogen-associated but also self-derived single-stranded RNA, and is associated with expression of IFN-inducible genes, such as *CXCL10*, through STAT1 activation (42). Moreover, TLR7 agonists were demonstrated to drive lupus nephritis in murine models (43) and, conversely, TRL7 blockade protects from the disease (44). Importantly, human CD14dim CD16+ monocytes, the likely executors of local surveillance and tissue patrolling, have been shown to be activated by viruses and immune complexes containing nucleic acids *via* a proinflammatory TLR7/TLR8-MyD88-MEK pathway (45). Interestingly, we observed a similar selectively augmented response to TLR7/8 ligands by the STAT1 GOF myeloid DCs and we suspect it to be due to the same unknown mechanism shared by the mutated myeloid lineage. Similarly to monocytes, TLR7 hyperactivity in mDCs was previously suggested to drive lupus nephritis in murine models (46). Thus, we propose that monocytes (and DCs) contribute to the pathogenesis of STAT1 GOF-associated autoimmunity, which may be at least partly driven by the TLR7/8 hyperresponsiveness to self-nucleic acids.

The interpretation of the hereby observed skew of the subtypes of STAT1 GOF DCs is largely hypothetical, as only limited understanding currently exists regarding functions and biology of individual DCs subsets. For example, the diminished pool of pDCs, characterized by their antiviral sensing and interferon production, may contribute to the viral susceptibility of STAT1 GOF patients. The pDCs’ decrease may be caused by type I IFN negative feedback loop (47), potentiated by type I interferon milieu in STAT1 GOF (correspondingly, IFNα was increased in the sera of our patients). The increase of DC4 subset, also known for its involvement in antiviral immunity and interferon response (24), may then be a secondary compensatory mechanism, and also contribute to the enhanced IFN signature. Moreover, DC4 cells have been shown to express high amounts of TLR7/8 (48), again possibly contributing to the self-tolerance failure. The DC2 subset, expanded in our STAT1 GOF patients, may be an important player in shaping the T cell response. This subset was described to be an effective T cell activator in psoriasis with capacity to polarize Th17 (49). This is, however, puzzling because Th17 are, in fact, decreased in STAT1 GOF. More data are needed to determine the role of DCs STAT1 GOF and how the abnormal subsets translate into the disease.

Although we were unable to provide direct evidence that the phenotypical alterations observed in STAT1 GOF monocytes are mechanistically linked to the augmented STAT1 signaling, and not merely secondary to the overall chronic inflammatory milieu or salvage regulatory mechanisms, several indices would suggest so. Most importantly, we show that *in vitro* generated STAT1 GOF monocyte-derived DCs retain the abnormal features of primary patients’ monocytes, including increased surface expression of CD86, decreased expression of PD-L1 and CD16, and increased production of inflammatory cytokines. Additionally, chronic stimulation in monocytes was shown to result in cellular exhaustion characterized by reduction of CD86 and elevation of PD-L1 (50–52), which is contrary to the phenotype of STAT1 GOF monocytes observed by us.

In summary, the dataset presented here indicates a proinflammatory skew and abnormal functions of STAT1 GOF monocytes of patients with CMC, which may directly contribute to both their microbial susceptibility and autoimmunity. These observations instate monocytes as prominent co-orchestrators of STAT1 GOF-associated immune dysregulation. As our data suggest, the aberrations seen in monocytes are likely carried onto other monocyte-derived cells, which outlines and substantiates a direction of possible future research in STAT1 GOF towards the DCs or macrophage biology.

## Data availability statement

The original contributions presented in the study are included in the article/**Supplementary Materials**. Further inquiries can be directed to the corresponding author.

## Ethics statement

The studies involving human participants were reviewed and approved by Ethical Committee of the second Faculty of Medicine, Charles University in Prague, and University Hospital in Motol, Czech Republic. Written informed consent to participate in this study was provided by the participants’ legal guardian/next of kin.

## Author contributions

MB conceived the hypothesis, treated the patients and co-wrote the manuscript.

IZ design the study, performed experiments, analyzed and visualized data and reviewed the manuscript. TM and AS treated the patients, provided biological material and reviewed the manuscript. ZP conceived the hypothesis, designed the study, performed phosphoflow experiments, analyzed and visualized data, and wrote the manuscript. All authors contributed to the article and approved the submitted version.

## Acknowledgments

The study was funded by kind support from Jeffrey Model Foundation. We thank the patients and healthy volunteers for the blood samples used in this study. We confirm that this manuscript has not been published elsewhere and is not under consideration by another journal.

## Conflict of interest

The authors declare that the research was conducted in the absence of any commercial or financial relationships that could be construed as a potential conflict of interest.

## Publisher’s note

All claims expressed in this article are solely those of the authors and do not necessarily represent those of their affiliated organizations, or those of the publisher, the editors and the reviewers. Any product that may be evaluated in this article, or claim that may be made by its manufacturer, is not guaranteed or endorsed by the publisher.

## Supplementary material

Supplementary Figure 1
**(A)** Intracellular and extracellular expression of CD16 detected by flow cytometry.
