# Supplementary figures and images for: Immunoprofiling of monocytes in STAT1 gain-of-function chronic mucocutaneous candidiasis
Source: Front Immunol. 2022 Sep 12;13:983977. doi: 10.3389/fimmu.2022.983977 (PMC9510987; doi:10.3389/fimmu.2022.983977)

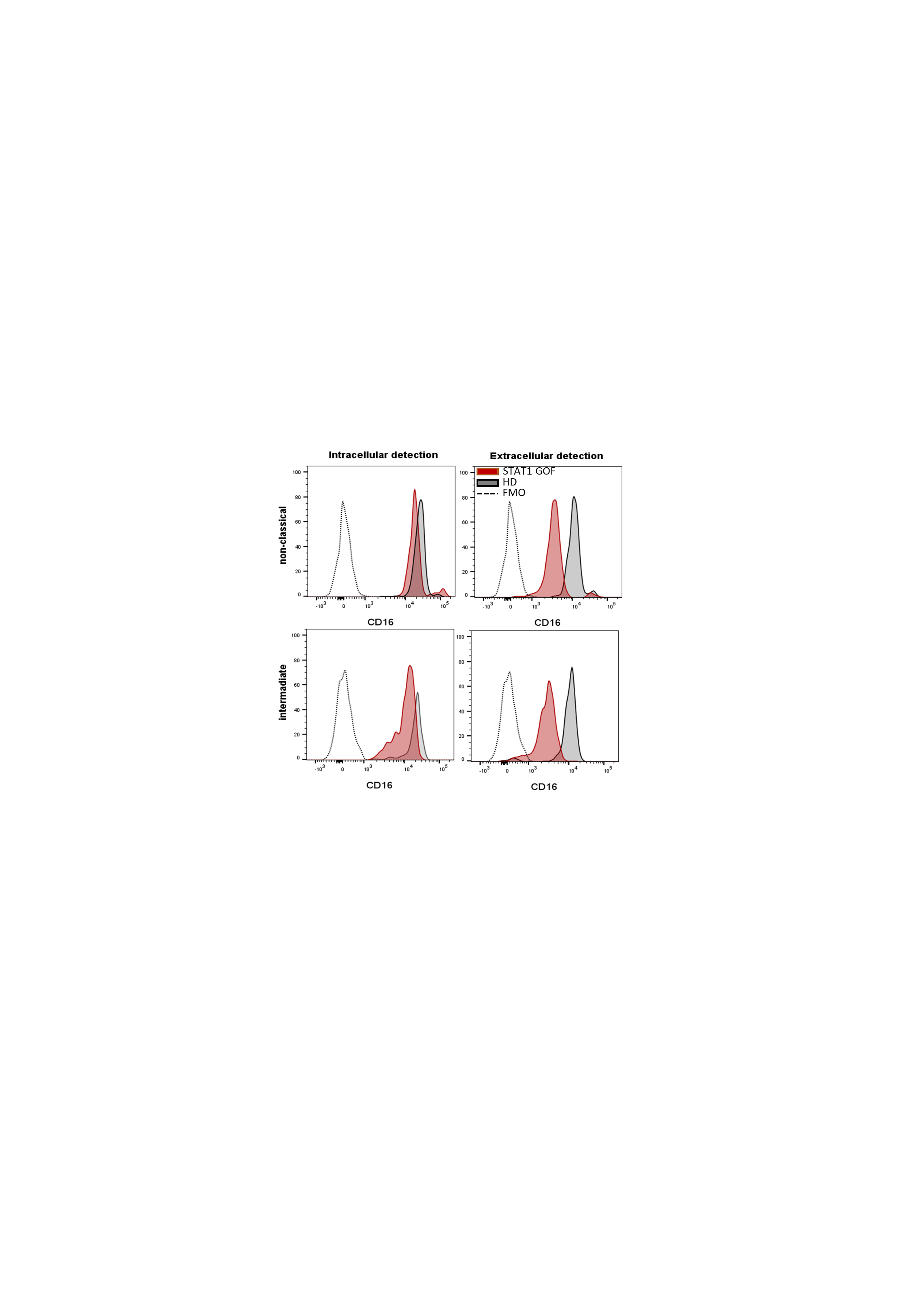

Supplement: Supplementary Figure 1 — (A) Intracellular and extracellular expression of CD16 detected by flow cytometry. [file Image_1.tif]
