# Supplementary material for: Immunoprofiling of monocytes in STAT1 gain-of-function chronic mucocutaneous candidiasis
Source: Front Immunol. 2022 Sep 12;13:983977. doi: 10.3389/fimmu.2022.983977 (PMC9510987; doi:10.3389/fimmu.2022.983977)
Supplement: Supplementary file 2 [file Table_1.docx]

**Supplementary Table: Genetic, immunologic and clinical characteristics of STAT1 GOF patients.**

a-AECA – anti-endothelial cell antibodies; a-CCP – anti-cyclic citrulinated peptide; a-EM – anti-endomysiu;, a-GLD – anti-gliadin antibodies; a-TG – anti-thyreoglobulin; a-TTG – anti-thyreoglobulin; ANA – anti-nuclear antibodies; ASCA – anti-saccharomyces cerevisiae; CRP – C-reactive protein; CMC - chronic mucocutaneous candidiasis; M/F – male/female; RF – rheumathoid factor; * = prior to immunoglobulin replacement therapy, where applicable; ↑= increased; ↓ = decreased;

° = prior to ruxolitinib, where applicable; Th17 = CD4+ IL17+; pSTAT1 = IFNγ/ IFNα -induced phosphorylation of p-STAT1 (Tyr701) in CD3+ T lymphocytes

|  | **cDNA position;**  **aminoacid change** | **Protein domain** | **M/F** | **Age (years)** | **CMC** | **Autoimmunity** | **Autoantibodies** | **Treatment** | **IgG*** | **IgA*** | **IgM*** | **T cells°** | **Th17°** | **B cells°** | **Neutrophil count°** | **Monocyte count°** | **CRP** | **pSTAT1° (Tyr701)** |
| --- | --- | --- | --- | --- | --- | --- | --- | --- | --- | --- | --- | --- | --- | --- | --- | --- | --- | --- |
| **P1** | c.86A>C; E29A | N-terminal | M | 45 | **+** | autoimmune anaemia  thyreopathy, hepatitis,  celiac disease | Coombs,  a-TTG, a-EM,  a-TG | azoles, ruxolitinib  previously corticosteroids, rituximab | N | N | N | N | **↓** | **↓** | N | N | **↑** | **↑** |
| **P2** | c.203A>G; Y68C | N-terminal | F | 21 | **+** | - | ASCA | azoles, immunoglubulin replacement therapy, ruxolitinib | N | **↑** | **↓** | N | N | N | N | N | **↑** | **↑** |
| **P3** | c.203A>G; Y68C | N-terminal | F | 45 | **+** | thyreopathy | ASCA, ANA,  a-GLD | azoles | **↑** | **↑** | N | N | **↓** | **↓ CD27+ memory** | N | N | **↑** | **↑** |
| **P4** | c.800C>T; A267V | Coiled-coil | F | 46 | **+** | thyreopathy | ASCA | azoles, trimethoprim | **↑** | **↓** | N | N | **↓** | N | N | N | N | **↑** |
| **P5** | c.863C>A; T288N | Coiled-coil | F | 52 | + | - | ASCA, CCP, AECA | azoles | N | N | N | **↓ CD4** | **↓** | **↓ CD27+ memory**  **↓ Class switched** | N | N | **↑** | **↑** |
| **P6** | c.1069A>G; N357D | DNA-binding | M | 8 | **+** | - | - | azoles | N | N | **↓** | N | **↓** | **↑ IgD^-^CD27^-^ naive** | N | N | N | **↑** |
| **P7** | c.1069A>G; N357D | DNA-binding | M | 45 | **+** | - | ASCA, RF | azoles | N | **↑** | N | N | **↓** | N | N | N | N | **↑** |
| **P8** | c.1169T>C; M390T | DNA-binding | F | 18 | **+** | - | - | azoles, amphotericin B, immunoglubulin replacement therapy, ruxolitinib | **↑** | N | N | **↓ CD4** | **↓** | N | N | N | **↑** | **↑** |
